# A quantitative tumor‐wide analysis of morphological heterogeneity of colorectal adenocarcinoma

**DOI:** 10.1002/2056-4538.70034

**Published:** 2025-06-13

**Authors:** Mihnea P Dragomir, Vlad Popovici, Simon Schallenberg, Martina Čarnogurská, David Horst, Rudolf Nenutil, Fred Bosman, Eva Budinská

**Affiliations:** ^1^ Institute of Pathology Charité‐Universitätsmedizin Berlin Berlin Germany; ^2^ German Cancer Consortium (DKTK), Partner Site Berlin, and German Cancer Research Center (DKFZ) Heidelberg Germany; ^3^ Berlin Institute of Health (BIH) Berlin Germany; ^4^ Masarykova Univerzita, RECETOX Brno Czech Republic; ^5^ Masaryk Memorial Cancer Institute Brno Czech Republic; ^6^ University Institute of Pathology University of Lausanne Lausanne Switzerland

**Keywords:** colorectal cancer, heterogeneity, morphology, AI‐based image analysis

## Abstract

The intertumoral and intratumoral heterogeneity of colorectal adenocarcinoma (CRC) at the morphologic level is poorly understood. Previously, we identified morphological patterns associated with CRC molecular subtypes and their distinct molecular motifs. Here we aimed to evaluate the heterogeneity of these patterns across CRC. Three pathologists evaluated dominant, secondary, and tertiary morphology on four sections from four different FFPE blocks per tumor in a pilot set of 22 CRCs. An AI‐based image analysis tool was trained on these tumors to evaluate the morphologic heterogeneity on an extended set of 161 stage I–IV primary CRCs (*n* = 644 H&E sections). We found that most tumors had two or three different dominant morphotypes and the complex tubular (CT) morphotype was the most common. The CT morphotype showed no combinatorial preferences. Desmoplastic (DE) morphotype was rarely dominant and rarely combined with other dominant morphotypes. Mucinous (MU) morphotype was mostly combined with solid/trabecular (TB) and papillary (PP) morphotypes. Most tumors showed medium or high heterogeneity, but no associations were found between heterogeneity and clinical parameters. A higher proportion of DE morphotype was associated with higher T‐stage, N‐stage, distant metastases, AJCC stage, and shorter overall survival (OS) and relapse‐free survival (RFS). A higher proportion of MU morphotype was associated with higher grade, right side, and microsatellite instability (MSI). PP morphotype was associated with earlier T‐ and N‐stage, absence of metastases, and improved OS and RFS. CT was linked to left side, lower grade, and better survival in stage I–III patients. MSI tumors showed higher proportions of MU and TB, and lower CT and PP morphotypes. These findings suggest that morphological shifts accompany tumor progression and highlight the need for extensive sampling and AI‐based analysis. In conclusion, we observed unexpectedly high intratumoral morphological heterogeneity of CRC and found that it is not heterogeneity *per se*, but the proportions of morphologies that are associated with clinical outcomes.

## Introduction

Morphological heterogeneity within colorectal adenocarcinoma (CRC) is a well‐recognized phenomenon. For instance, the WHO 2019 classification defines mucinous adenocarcinoma, signet ring cell type, and adenoma‐like adenocarcinoma as tumors in which a minimum of 50% of the lesion is composed of these specific morphologies [1]. However, in diagnostic practice, morphological heterogeneity is often insufficiently considered [2], both in terms of making a morphological diagnosis and in selecting tissue samples for molecular analysis. In addition, the definitive cut‐offs for different morphologies are often controversial and arbitrary, making it difficult to assess the prognostic significance of morphologic heterogeneity in CRC [2]. The morphological diagnosis of a tumor is, as a rule, based on the dominant histological pattern. Likewise, tumor molecular profiling is typically performed on tumor regions that have been macro‐ or microdissected or punched out from a single FFPE block or on fresh frozen tumor samples obtained from a single tumor region, without taking morphological heterogeneity into account [3, 4]. In addition, for fresh‐frozen specimens used in molecular studies, histopathology is often performed on an FFPE block from a different tumor region, and all information regarding morphology, tumor purity, and even diagnosis is derived from the different tumor region. Therefore, such common practices may lead to some discrepancies between molecular and histological data.

As a result, molecular taxonomies of CRC such as the Consensus Molecular Subtype classification [5] are mostly based on tissue samples, morphologically classified without taking intratumor heterogeneity into account. Our group [6] and later others [7] managed to replicate transcriptome‐based molecular classification of CRC using image‐based surrogate analysis of readily available high‐resolution digital H&E sections. These results confirmed the close relationship between tumor morphology and coding transcriptome. Transcriptomic profiling of macrodissected morphologically distinct tumor regions, and comparison of the results with the transcriptome of a morphologically heterogeneous sample of the same tumor showed that these do not match [8]. These observations suggest that molecular profiling and classification of CRC should be based on morphologically well‐defined tumor tissue samples.

In this study, we investigated intratumoral morphological heterogeneity in CRC by addressing the following questions: (1) what is the extent of intratumoral morphological heterogeneity across CRC?; (2) are different morphologies topographically related?; and (3) does morphological heterogeneity impact clinical variables and CRC prognosis? To address these questions, prevailing morphotypes were established in four different tumor blocks per CRC by visual assessment by expert gastrointestinal pathologists and in parallel by an AI‐based tool. The main research objective of the study was to analyze and quantify the morphological intratumoral heterogeneity in CRC and to test potential associations between different morphotypes and clinical parameters.

## Materials and methods

### Sample collection and preparation

The experimental design of this study was retrospective. We used histopathological material from 161 consecutive histologically confirmed stage I–IV CRCs, retrieved from the hospital cohort of Masaryk Memorial Cancer Institute in Brno, Czech Republic. Tumors from patients who underwent neoadjuvant treatment and patients with multiple tumors were excluded. Initial diagnostic H&E evaluation was performed by an expert pathologist according to the 2019 WHO classification [1]. For each tumor, we determined the T‐stage, grade (G), localization, number of positive lymph nodes and total number of lymph nodes (N), and microsatellite instability (MSI) status.

For each case, in a standardized fashion, we selected four FFPE blocks representing: (1) the deepest invasion point at the serosa side (relation to serosa); (2) the deepest invasion point at the insertion side of mesocolon/mesorectum (relation to adipose tissue); (3) the transition point between the tumor and normal mucosa (including luminal side of the tumor); and (4) a central tumor block containing representative histology as far as possible. In this way, we wanted to get a global view of tumor heterogeneity. Of each of the 644 blocks a 5‐μm section was stained with H&E. The stained sections were scanned using the Pannoramic Midi (3DHISTECH, Budapest, Hungary) scanner at ×20 magnification (0.234 μm/pixel resolution), with the same settings across all scans.

The study was conducted in accordance with the principles of the Declaration of Helsinki. The study was approved by the ethical committee of Masaryk Memorial Cancer Institute and all patients provided written informed consent prior to enrolment.

### Morphotypes and heterogeneity analysis

Morphotypes were defined as frequently occurring ‘pure’ histological patterns in CRC as described in our previous publication [9]. These overlap in part with the carcinoma subtypes as defined in WHO 2019 [1]. We decided to use the term morphotypes to indicate a single histological pattern, whereas the WHO subtypes are (inherently) morphologically heterogeneous. The six morphotypes were: complex tubular [CT; as found in WHO adenocarcinoma NOS (non‐otherwise specified)]; solid/trabecular (TB; as found in WHO medullary and undifferentiated adenocarcinoma); mucinous (MU; as found in WHO mucinous adenocarcinoma, but without a 50% threshold); papillary (PA; mainly as found in WHO adenoma‐like adenocarcinoma); desmoplastic (DE; no WHO histopathological equivalent but defined as with a stromal component exceeding 50% of the tumor volume); and serrated (SE; as found in WHO serrated adenocarcinoma). As other morphological patterns encountered in WHO histopathological subtypes, such as signet‐ring cell carcinoma, adenosquamous carcinoma, and carcinomas with sarcomatoid components, are rare these were not considered in our morphotype definitions. The most frequently found morphotype was called ‘dominant’, the second most found as ‘secondary’ and the third most found as ‘tertiary’.

To define a training set for AI analysis, three different pathologists assessed morphotype presence on a selection of 22 cases in which at least two morphotypes had been identified. Morphotypes had been previously defined by one of us (FB) and digitally documented as photomicrographs. Each pathologist (MPD, SS, and RN) independently scored the defined morphotypes and no common training sessions were conducted to create ‘common ground’ in classifying. We used this pilot phase to define interobserver variability and to define regions with a morphotype on which all pathologists agreed (ground truth).

Furthermore, one of the expert pathologists (MPD) conducted a second assessment of the AI image analysis, determining whether the annotations were accurate (i.e., whether they represented a genuine phenomenon or a hallucination) and identifying their locations across the colonic wall (mucosa, submucosa, muscularis, and fat tissue).

### AI image analysis

In the training phase, a deep learning image analysis AI model (DenseNet V2) was trained to automatically detect the six morphotypes, using HALO® Image Analysis Platform (version 3.6.4134. Indica Labs, Inc., Albuquerque, NM, USA) and HALO® AI software (version 3.6.4134. Indica Labs, Inc.). The model was trained using eight digital section files at low magnification (equivalent to ×1.25), selected from the 22‐tumor set. The regions selected for training (from these sections) corresponded to regions with perfect agreement between expert pathologists and covered all six morphotypes. The training stopped once the agreement between annotations and predictions (defined as a modified ‘intersection‐over‐union’ coefficient: $\frac{|P\cap A|}{|A|}$, where *P* was the predicted region and *A* the annotated region), was greater than 0.9 for all categories of interest. Since the training procedure used transfer learning, a limited number of images was enough for reaching good performance. To reduce bias due to small regions that have a higher likelihood of being misclassified, we kept only the predicted regions occupying at least 5% of the tumor area in the section.

In the second phase, the AI model was applied to all 644 digital section files in the collection from the 161 CRC. AI automatically segmented all samples, annotated them for the presence of the six morphotypes, and quantified the surface area occupied by each morphotype. We also studied correlations between the morphotype characteristics, as a parameter of intratumoral heterogeneity, with clinical and pathological parameters.

### Statistical analysis

To measure inter‐rater reliability between the three pathologists, we calculated pairwise the Cohen's kappa coefficient for the observation of each of the morphotypes separately, regardless of their prevalence.

For certain analyses, tumor morphotype proportions/areas were defined as the average of proportions of individual sections. To quantify morphotype heterogeneity within each section and each tumor, we computed the Shannon index (standard measure of sample diversity, based on proportions of constituting parts) and standardized this index by dividing the observed values by the maximum possible value (1.79 for six uniformly distributed morphotypes), resulting in an index between 0 and 1 [normalized Shannon index (NSI); the value 0 indicates that the slide is 100% composed of one morphotype only and value 1 indicates that the slide is composed of equal – 16.67% – proportions of all six morphotypes]. NSI was computed either in each section separately or on the average of proportions across sections in the tumor (hereinafter tumor NSI).

To assess the association of clinical categorical variables (gender, TNM, stage, grade, site, MSI status) with the morphotype categories (present/absent) or between the two cohorts, Pearson's chi‐square test was employed. The differences in the tumor morphotype area between groups were assessed using Kruskal–Wallis test (for more than two categories) or Mann–Whitney *U*‐test (for two categories). Age was tested between the two cohorts using Student's *t*‐test.

Associations between morphotypes and overall and relapse‐free survival, in the whole cohort and in stage I–III patients only, were evaluated using Kaplan–Meier survival curves stratified by morphotype proportion. Optimal cut‐off values for dichotomization were determined in an exploratory analysis using regression survival trees (rpart package in R), which identified the most informative splits with respect to survival. Group differences in survival were assessed using the log‐rank test. Where appropriate, we corrected for multiple hypothesis testing by controlling false discovery rate (FDR) using the Benjamini‐Hochberg method. Results were considered significant at FDR <10%. All statistical analyses were performed using R version 4.3.2 [10]. Visualizations were performed using R packages ggplot2 (3.4.4), ggstatsplot (v.0.12.1), corrplot (v 0.92), and forestploter (v.1.1.1).

## Results

### Visual morphotype assessment

**Figure 1:**
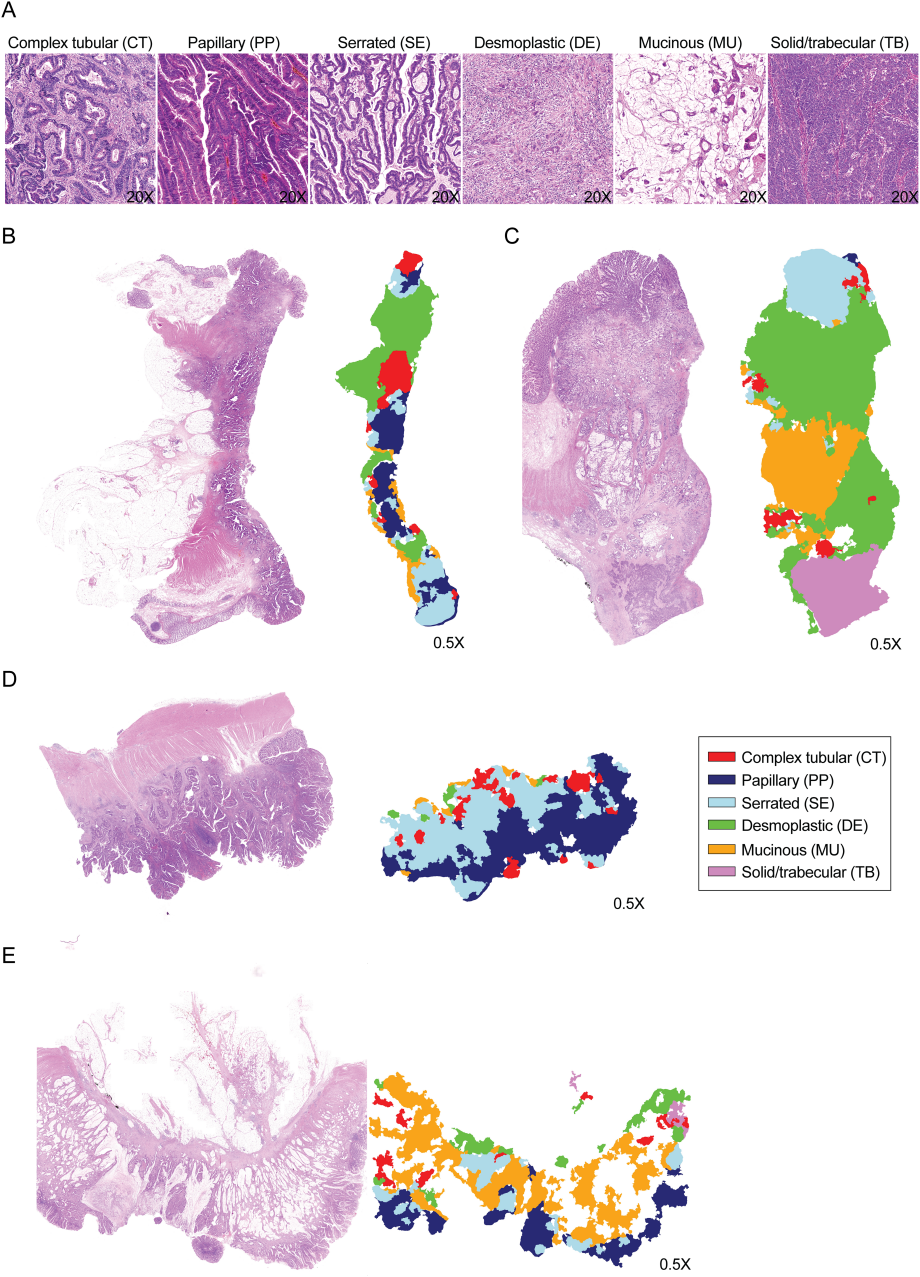
Visual and AI‐based morphotype assessment. (A) Representative H&E images of the six colorectal cancer morphotypes analyzed. All images are at a ×20 magnification. (B–E) H&E images of prototypical examples of morphotypes included in the study side by side with the morphotypes predicted by the AI image analysis tool. All images are at a ×0.5 magnification.

To test whether morphological analysis can be implemented in clinical practice and to generate the ground truth for a machine learning tool that can automatically detect morphotypes of CRC, three expert histopathologists evaluated the presence and distribution (as dominant, secondary, and tertiary, in terms of the proportion in the tumor section) of the six previously described morphotypes (Figure 1A) in four histological sections from different blocks of 22 tumors, matched for age, gender, site, and stage (Table 1).

**Table 1:** Distribution of clinical parameters of the individuals in the study, comparing the image analysis and pathologist sets

	Full set (image analysis)	Subset (pathologists evaluation)	*p*
*n*	161	22	
Age at diagnosis (years) [mean (SD)]	66 (11)	61 (14)	0.055
Gender = M (%)	89 (55.3)	11 (50)	0.812
Stage (%)			0.093
I	24 (14.9)	0 (0.0)	
II	67 (41.6)	10 (45.45)	
III	43 (26.7)	10 (45.45)	
IV	27 (16.8)	2 (9.1)	
Grade (%)			0.170
1	19 (11.8)	5 (22.7)	
2	95 (59.0)	14 (63.7)	
3	47 (29.2)	3 (13.6)	
Site (%)			0.038
Right	55 (34.2)	11 (50.0)	
Transverse	18 (11.2)	0 (0.0)	
Left	44 (27.3)	10 (45.5)	
Rectosigmoid	27 (16.8)	1 (4.5)	
Rectum	17 (10.6)	0 (0.0)	
pT stage (%)			0.465
T1	8 (5.0)	1 (4.5)	
T2	25 (15.5)	1 (4.5)	
T3	115 (71.4)	19 (86.5)	
T4	13 (8.1)	1 (4.5)	
pN stage (%)			0.478
N0	94 (58.4)	10 (45.5)	
N1	41 (25.5)	8 (36.4)	
N2	26 (16.1)	4 (18.2)	
pM stage = M1 (%)	27 (16.8)	2 (9.1)	0.539
MS‐Status = MSS (%)	104 (79.4)	11 (90.9)	0.598

The pathologist set is a subset of the image analysis set.

For CT, PP and SS, the interobserver variability was high, while it was lower for MU, DE, and TB (supplementary material, Figure S1). All pathologists scored CT as the most frequently present morphotype in all sections and in tumors (supplementary material, Figure S2A,B). The pathologists disagreed on the least frequent morphotype: TB, DE, and SE (supplementary material, Figure S2A,B). In tumors containing the CT morphotype, it was dominant in at least one of the tumor sections in most tumors (supplementary material, Figure S2C). DE was never the dominant morphotype in all four sections of a tumor (supplementary material, Figure S2C). In most tumors, two or three different morphotypes were dominant across the four sections (supplementary material, Figure S2D). In most tumors, two to four different secondary morphotypes and more than two different tertiary morphotypes were found (supplementary material, Figure S2D). Overall, while the recognition of the six morphotypes showed an acceptable degree of inter‐pathologist reproducibility, the semi‐quantitative categorization showed high inter‐pathologist variability.

Next, to obtain an objective quantitative basis per case, we developed an AI‐based image analysis tool. From this 22‐tumor set, regions with perfect agreement between expert pathologists that covered all six morphotypes were selected for the AI model training. Representative annotations of the AI‐based image analysis results side by side to prototypical H&E slides can be found in Figure 1B–E.

### Morphotype assessment by AI

**Figure 2:**
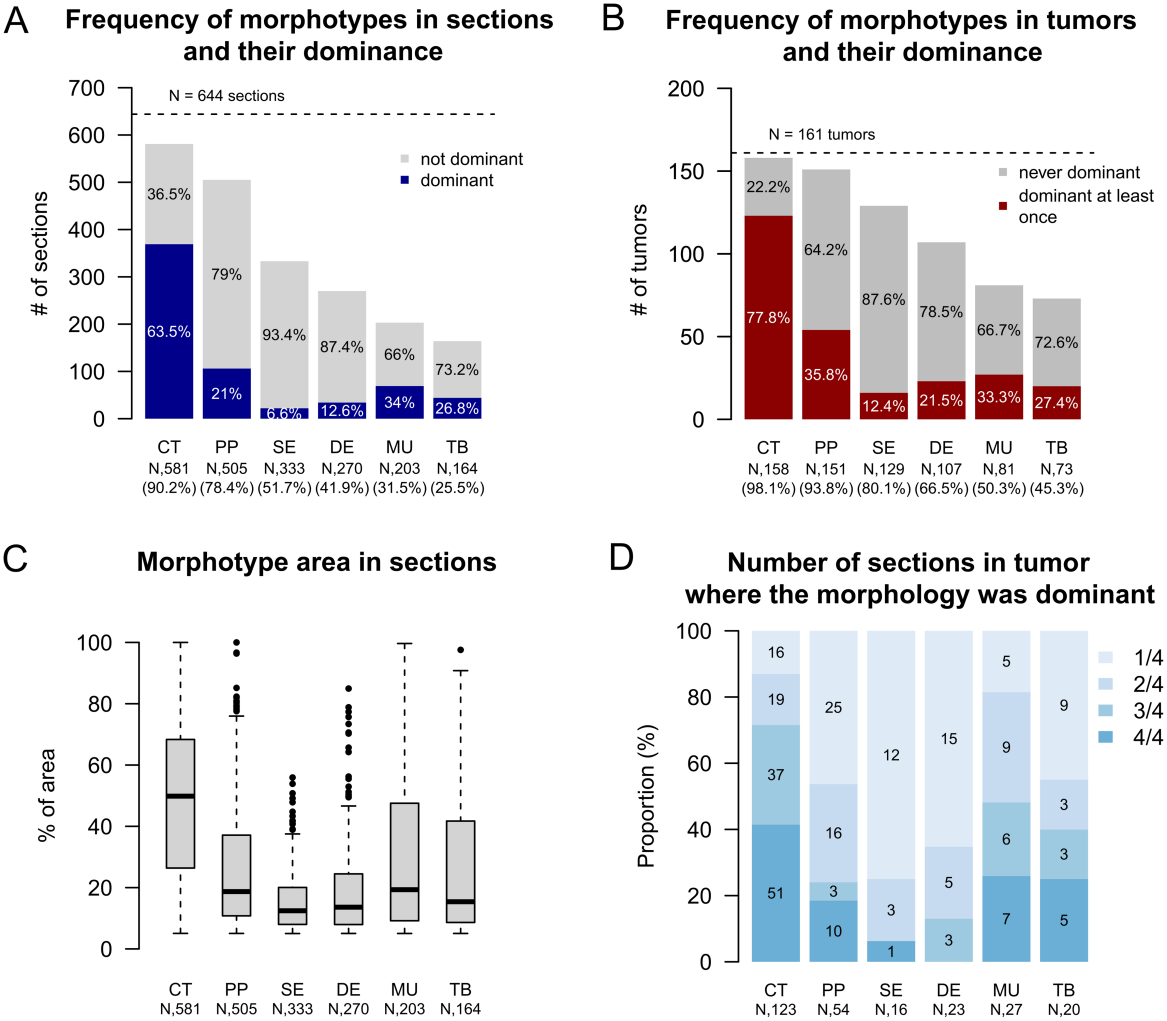
Frequency and area of dominant morphotypes across the examined sections and tumors. (A) Frequency of morphotypes in sections with respect to their dominance. (B) Frequency of morphotypes in tumors with respect to their dominance. (C) Distribution of the fragment areas per individual morphotype, across all the sections. (D) Frequency of sections in tumors where the morphology was dominant. 1/4 means the morphotype was dominant in one of the four examined slides of the tumor *etc*.

The clinicopathological characteristics of the set of 22 cases evaluated by pathologists were quite similar to the full set of 161 cases; the only significant differences were regarding tumor site (Table 1). AI‐based image analysis allowing automated reproducible quantification of percentage of area per morphotype per section was applied to the 644 digital section files. By AI, CT was again the most commonly present morphotype in individual sections (581/644, 90.2%) as well as in the combined sections of a tumor (158/161, 98.1%). The least common morphotype was TB (in sections 164/644, 25.5% and in tumors 73/161, 45.3%) (Figure 2A,B). CT morphotype also tended to occupy the largest area of tumor within a section (mean 49.8%); for other morphotypes this was between 12.5% (SE) and 19.3% (PP) (Figure 2C). CT morphotype was the dominant morphotype in at least one of the four tumor sections in most tumors (123/158, 77.8%) (Figure 2B). Other morphotypes were rarely dominant (Figure 2B,D). DE was never the dominant morphotype in all four examined sections of a tumor but most often dominant in only one of four sections (15/23, 65%), similar to SE (12/16, 75%) (Figure 2D).

Two or three different dominant morphotypes were found across the four examined H&E sections in 54% (87/161) of the tumors; four different dominant morphotypes were never observed in a tumor. Two to four secondary morphotypes were found in 86.3% (139/161) tumors and two to four tertiary morphotypes in 74.5% (120/161). Overall, these initial observations match well with those of the expert pathologists.

### In most tumors, intratumoral morphological heterogeneity is average or high

**Figure 3:**
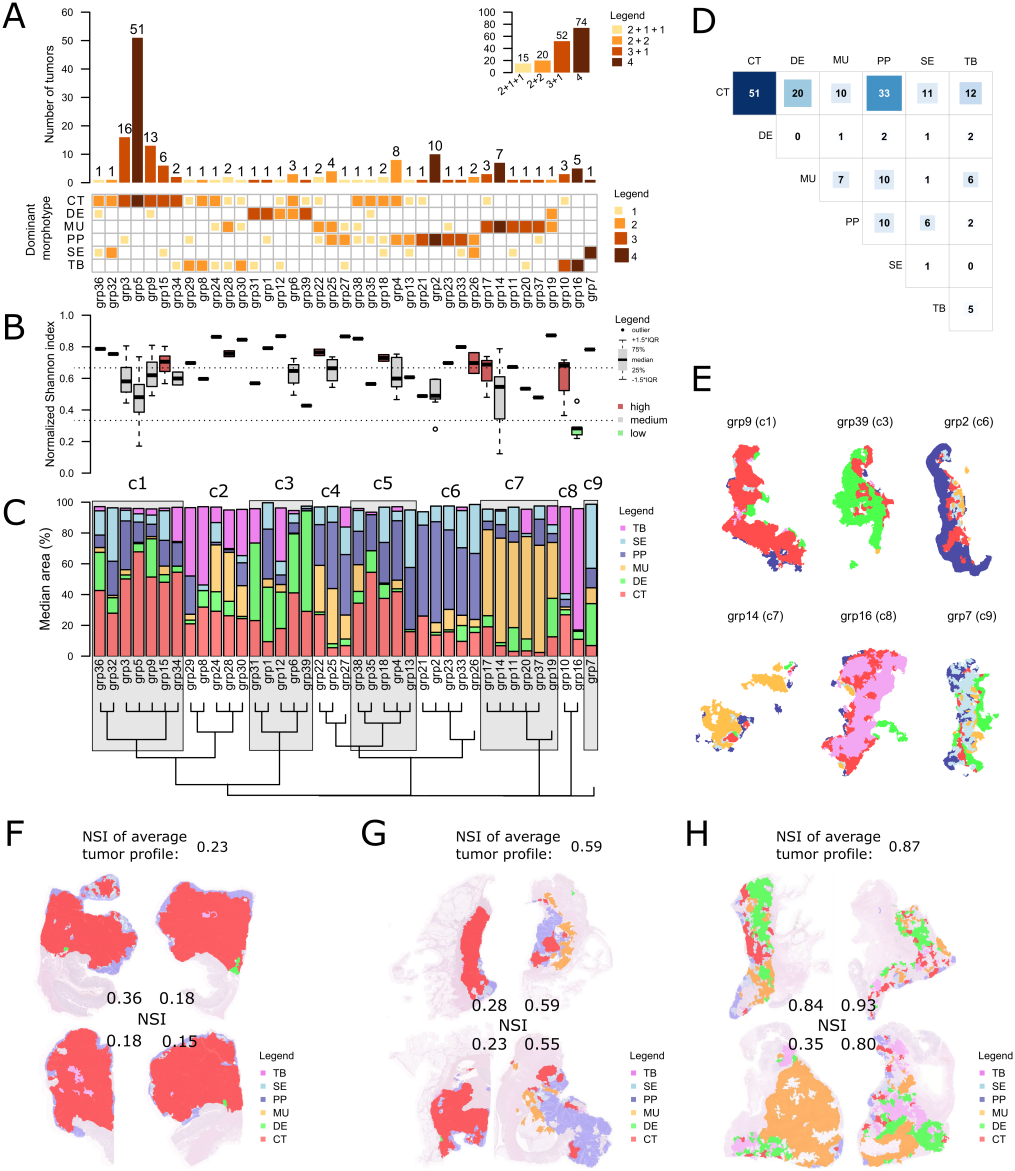
Patterns of intratumoral morphological heterogeneity. (A) Observed intratumoral patterns of dominant morphotype combinations (IPDMCs) and their frequency (main barplot) and frequency of their distribution patterns (embedded top right barplot). (B) Distribution of normalized Shannon index (NSI) of median tumor profiles in the IPDMCs. (C) Median morphotype area in the IPDMCs and their further clustering into nine clusters. (D) Frequencies of pairwise combinations of dominant morphotypes in the sections. (E) Examples of representative tumor morphological areas of slides from selected IPDMCs clusters as identified by image analysis. (F–H) Examples of intratumoral morphological heterogeneity as assigned by image analysis over four examined slides/blocks. Values of NSI of each slide and the average tumor profile are shown. (F) Tumor with low heterogeneity across all slides, expressing one dominant morphotype (CT). (G) Tumor with low heterogeneity in two slides and medium heterogeneity in two slides, expressing two dominant morphotypes (CT and PP). (H) Tumor with high heterogeneity in all four sections, expressing two dominant morphotypes (DE and MU).

Figure 3 documents the striking degree of intratumoral heterogeneity. When distribution patterns (4, 3 + 1, 2 + 2, and 2 + 1 + 1) of dominant morphotype combinations (DMC) in the four sections per tumor are plotted, 39 groups (grp) emerge (Figure 3A). The most frequent DMCs were 3 × CT + 1 × PP (*n* = 16 tumors), followed by 3 × CT + 1 × DE (*n* = 13), 2 × CT + 2 × PP (*n* = 8), and 3 × CT + 1 × SE (*n* = 6).

We further evaluated intratumoral morphological heterogeneity by computing a normalized Shannon diversity index (NSI, range 0–1) – this is a well‐established diversity measure based on the proportions of constituent parts. NSI was computed on individual sections as well as on the average tumor proportions (defined as the mean of proportions of each morphotype across the four sections of the tumor). This proportion standardizes the potential effect of the number of sections. We visualized NSI values of the average tumor proportions for each DMC group (Figure 3B). In most tumors, NSI varied around the median (between 0.3 and 0.7) or was in the higher range (NSI > 0.7), while NSI was in the lower range in only a few tumors (NSI < 0.3 group 16; Figure 3B); the latter were composed mostly of solid/trabecular morphotypes.

Hierarchical clustering of the tumor morphotype proportions further divided the DMCs into nine clusters (Figure 3C). Associations and example sections of the DMCs can be found in Figure 3D,E. Figure 3F–H shows examples of all four sections from tumors with low, medium, and high morphological heterogeneity as defined by their NSI.

### Tumor morphotype proportion is correlated with pathological and clinical parameters

Finally, having the variables at hand, we also explored clinical and pathological parameters that were associated with the morphotypes (expressed as the average proportion of the morphotype area in the tumor) and the degree of morphotype heterogeneity (expressed as the tumor NSI and as distribution patterns of the DMCs).

No significant associations were found between tumor NSI and clinical parameters, nor between the distribution patterns of DMCs across stages (I–IV, Pearson's chi‐squared test, *p* = 0.4561) or tumor site (left versus right, Pearson's chi‐squared test, *p* = 0.151).

**Figure 4:**
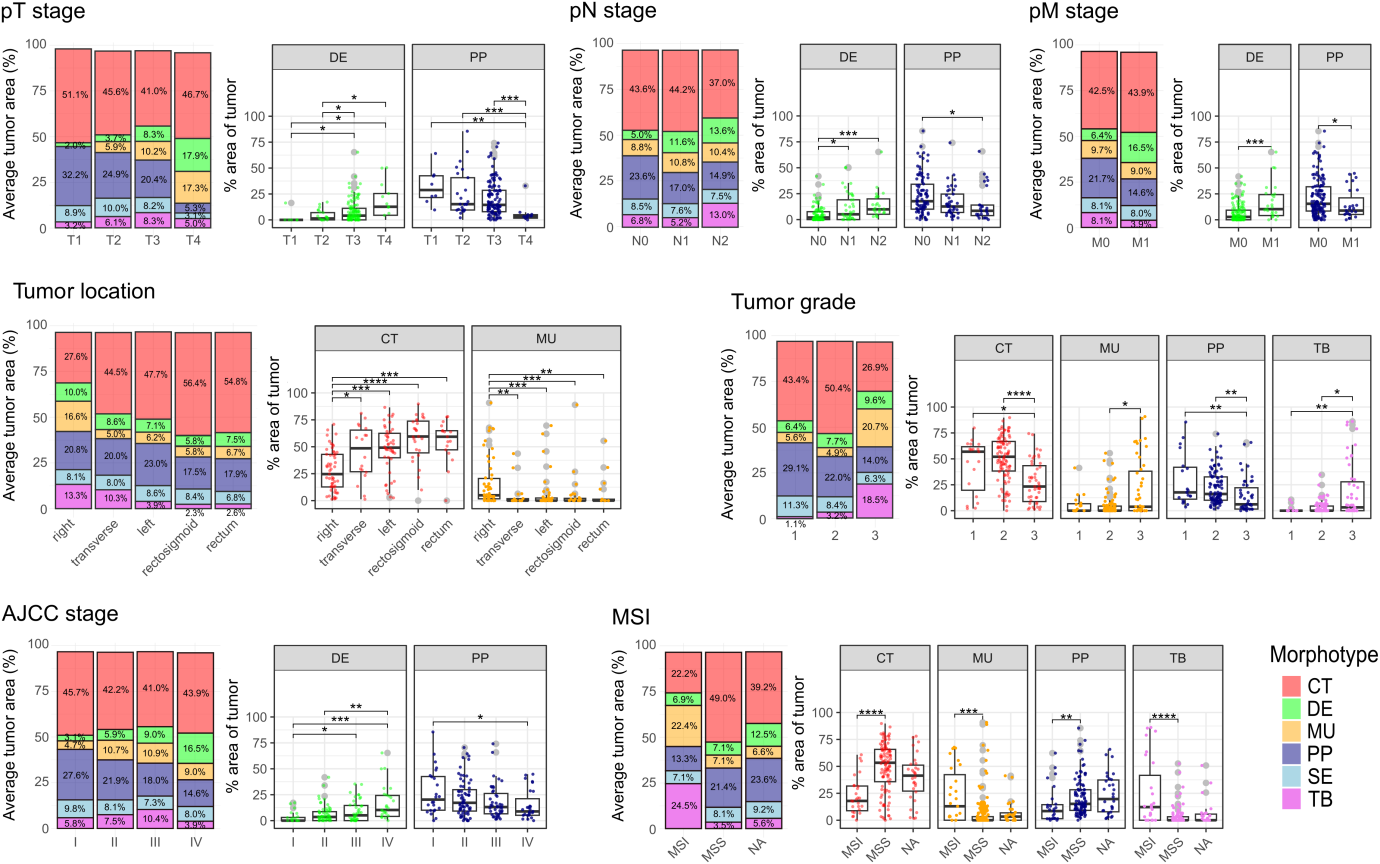
Associations between clinical variables and the tumor morphotype area. For each clinical variable, two types of graphs are shown. Left: a stacked barplot representing the average tumor area profile per category. Right: box plots of significant associations of tumor morphotype area with categories of clinical variables, where the middle line represents the median, the box represents the interquartile range (IQR), the whiskers represent ±1.5 × IQR, and the gray dots represent outliers. Colored dots represent single values per tumor; significant associations between the respective categories are indicated by connected lines and marked as follows: *p < 0.05; **p < 0.01; ***p < 0.001; ****p < 0.0001 (Mann–Whitney *U*‐test between pairs of categories, if Kruskal–Wallis test significant). Additional associations and full statistics are provided in supplementary material, Table S1.

An increase in the proportion of the DE morphotype and a decrease in the proportion of the PP morphotype were associated with higher T‐stage, N‐stage, AJCC‐stage, and the presence of synchronous distant metastases (Figure 4 and supplementary material, Table S1). The proportion of MU or TB morphotypes was associated with higher grade, right side, and MSI. Notably, the MU morphotype was absent in all tumors classified as stage T1. The proportion of the CT morphotype was significantly higher in left‐sided tumors including rectosigmoid and rectum. CT and PP proportions were significantly lower in grade 3 and MSI tumors (Figure 4). No significant associations were observed between the SE morphotype and any of the clinical variables (supplementary material, Table S1).

**Figure 5:**
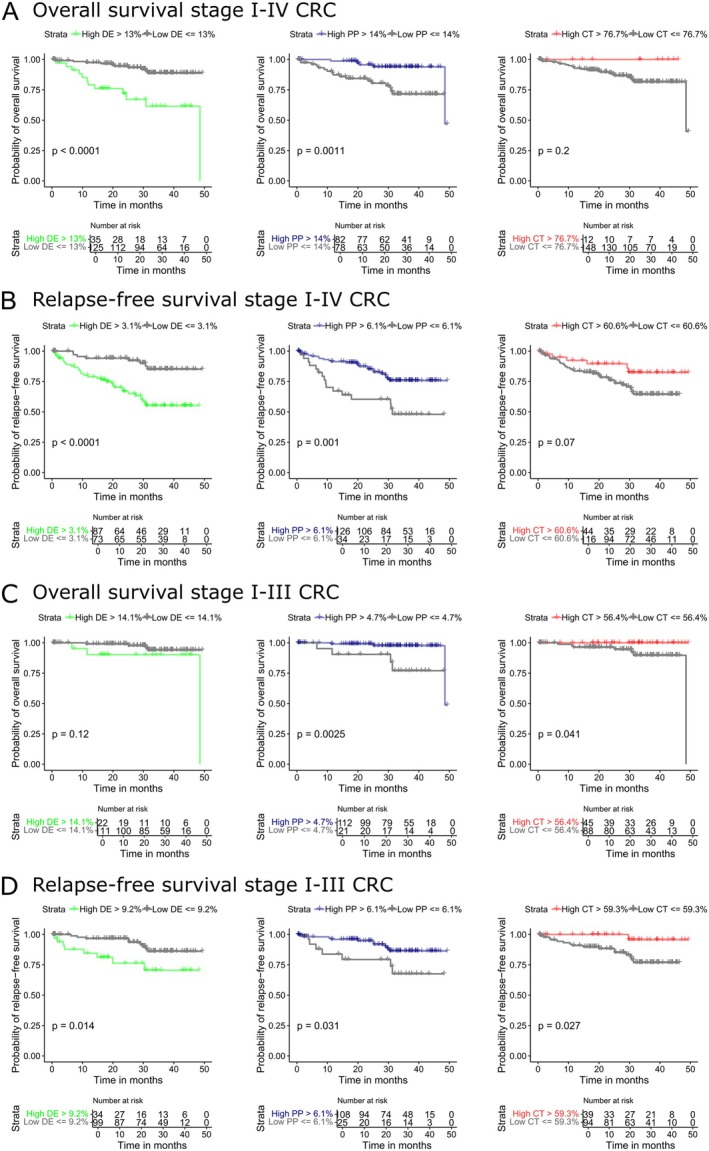
Kaplan–Meier survival curves and risk tables for key morphotypes. Kaplan–Meier curves depict overall survival (OS) and relapse‐free survival (RFS) stratified by the proportion of desmoplastic (DE), papillary (PP), and complex tubular (CT) morphotypes. Optimal cut‐offs for high versus low morphotype proportion were determined using regression survival trees. (A) OS in the full cohort. (B) RFS in the full cohort. (C) OS in stage I–III tumors. (D) RFS in stage I–III tumors. Risk tables indicate the number of patients at risk at each time point. Differences between groups were assessed using the log‐rank test.

Finally, we investigated possible associations between the tumor area of individual morphotypes as well as tumor NSI and overall survival (OS) and relapse‐free survival (RFS) in all the cohort and only in stage I–III CRC patients. Optimal cut‐off values for dichotomization were determined in an exploratory analysis using regression survival trees. In the whole cohort, a higher proportion of the DE morphotype was significantly associated with shorter OS (*p* < 0.0001; Figure 5A) and RFS (*p* < 0.0001; Figure 5B). In the stage I–III subgroup, this association was lost for OS (*p* = 0.12; Figure 5C) but remained significant for RFS (*p* = 0.014; Figure 5D). An increased proportion of the PP morphotype correlated with longer OS and RFS in both the whole cohort (*p* = 0.0011 and *p* = 0.001, respectively; Figure 5A,B) and the stage I–III subgroup (*p* = 0.0025 and *p* = 0.031; Figure 5C,D). Lastly, a higher proportion of the CT morphotype was associated with longer OS (*p* = 0.041; Figure 5C) and RFS (*p* = 0.027; Figure 5D) in the stage I–III subgroup only; no significant associations were observed in the full cohort. No significant association was found between NSI and survival. Due to the limited numbers, we did not perform survival analysis between the DMC groups.

### Reassessment of AI by pathologists

Finally, the findings of the AI‐based image analysis for the 22 initial tumors were conveyed to one of the expert pathologists (MPD). There was a high level of concordance between the AI and the expert pathologists regarding the primary, secondary, and tertiary morphotypes, with only a few instances of *bona fide* AI hallucination identified. The expert pathologists agreed with the AI in 97.22% of the cases (280/288), and the hallucinations were mainly detected for small areas/tertiary morphotypes.

In addition, the expert pathologist analyzed the AI labeling by determining the intratumoral across‐the‐colonic‐wall location of the 288 dominant, secondary, and tertiary morphotypes. CT was predominantly located in the submucosa and muscularis propria (superficial), DE primarily in the muscularis propria and fat tissue (deep), MU in the submucosa and muscularis propria (mixed, both superficial and deep), PP in the mucosa (superficial), SE in the mucosa and submucosa (superficial), and TB in the muscularis propria (deep) [chi‐square test, after combining mucosa with submucosa (superficial) versus muscularis propria and fat tissue (deep), *p* < 0.0001; supplementary material, Figure S3].

## Discussion

Our results confirm that CRC histology is highly heterogeneous, both between tumors as well as intratumoral. Intratumoral heterogeneity was even more striking when four sections from different FFPE blocks of the tumors were examined. A pilot analysis by expert pathologists on 22 selected cases using the morphotype concept allowed us to define morphotypes reproducibly recognized by the experts but also showed that inter‐pathologist reproducibility of the semi‐quantitative assessment of dominant, secondary, and tertiary morphotypes was insufficient. Interobserver variability regarding the morphological assessment of CRC is well known [11]. We therefore identified sections on which the pathologists agreed regarding the dominant morphotype and used these to train an AI tool to identify the presence of the defined morphotypes per tumor and to quantify the proportion of each morphotype in individual sections as well as in the four combined sections per tumor. The quantitative data obtained were used to assess the potential clinical relevance of the identified (heterogeneous) morphological patterns.

Both pathologist's visual assessment and AI showed that, in more than 50% of tumors, the dominant morphotype is different between different tumor sections. As morphotypes are the leading criteria for histological (sub)typing of CRC, the direct implication is that a (sub)type should never be called on a single section, which corresponds with common practice as the guidelines in use all define the number of tissue samples to be taken, invariably exceeding our number of four. Our use of the Shannon index, which combines proportions of the different morphotypes, showed that most tumors display a degree of heterogeneity in the middle to higher range. Tumors with a low degree of heterogeneity appeared to be mostly of the TB morphotype. This corresponds to a poorly differentiated adenocarcinoma with a loss of glandular or villous architecture. We also found that the CT morphotype, which corresponds to conventional adenocarcinoma NOS [12], is the most prevalent and hence is found in combination with all other morphotypes. When present, this morphotype is also most often dominant. In approximately 30% of cases, the SE morphotype dominated, indicating involvement of the serrated neoplasia pathway [13]. Other associations between morphotypes and CRC oncogenesis pathways are the PP and MU morphotypes defining adenoma‐like and mucinous carcinomas, which are enriched in *KRAS* or *BRAF* driver mutations [12, 14, 15, 16].

Currently, molecular pathology of CRC focuses on driver genes which determine the relevance of therapy targeting EGFR and on microsatellite status, in the setting of recurrent and metastatic disease [17] and heterogeneity is disregarded. Büttner *et al* reported two cases in which the *KRAS* status differed between histotypes. In one case, mucinous morphology was WT for *KRAS*, while non‐mucinous morphology showed the *KRAS* G12D mutation. A second case showed a *KRAS* G12D mutation in non‐mucinous morphology and a targetable *KRAS* G12C mutation in a mucinous region [18]. This leads to the question of whether different morphotypes should be sampled for molecular analysis and how data documenting molecular heterogeneity should be used in decision making regarding therapeutic approaches. This is also true for transcriptional data supporting molecular subtypes of CRC that have not yet entered clinical practice. CRC cases with CMS4 subtype have been reported to receive limited benefit from standard chemotherapy like oxaliplatin and 5‐fluorouracil or anti‐EGFR therapy, despite being *KRAS* WT [19, 20, 21, 22]. We contend that further studies on correlations between morphotype and genomic or transcriptomic characteristics are needed to arrive at evidence‐based selection of the most relevant tissue sample(s) for molecular analysis.

Earlier studies have emphasized the conceptual and potential clinical importance of morphological heterogeneity in CRC [16]. The CMS subtypes of CRC, which have prognostic and predictive implications [5] and hence have entered the realm of clinical trials, were established using tumor tissue samples without due attention to intratumor heterogeneity, even though the potential relevance of intratumoral heterogeneity was recognized [5]. Current methods, such as AI‐based analysis of H&E sections [23] or spatial transcriptomics [24, 25, 26], offer the possibility to study the association between morphological and molecular heterogeneity of CRC *in situ*. Such studies have already confirmed the high degree of intratumoral heterogeneity, and new links between morphology and underlying molecular programs have been established [6, 7, 24]. Our study shows that, for meaningful molecular analysis, a tissue sample from one section/block may not be sufficient. In addition, generic (sub)typing of the tumor is not enough: a tissue sample for molecular analysis should be characterized in terms of morphotype. Many departments of pathology are moving to full digital workflow. We postulate that an AI‐based morphotype analysis could introduce a quantifiable dimension to morphology, potentially enhancing the histopathological characterization of CRC over time.

We did not find any association between the degree of intratumoral heterogeneity and clinical outcomes, but we found correlations between single morphotypes and clinical and pathological parameters, providing further evidence of the potential importance of quantifying specific morphotypes. We found tumors with low CT proportion to be more often MSI, and a high proportion of MU morphotype to correlate with MSI and right‐sided location, as has been reported before [12, 27]. The association between an increased proportion of DE and higher T‐stage and local and distant metastases, as well as shorter RFS, fits with the published association between the desmoplastic reaction and prognosis [28]. However, the desmoplastic reaction has been subcategorized into mature, intermediate, and immature, the immature form having the worst prognosis [29], and our morphological approach did not make this subclassification. Furthermore, these associations can be partially attributed to the fact that the DE morphotype is typically situated at the tumor front and within the deeper layers of the colon wall, including the muscularis propria and adipose tissue. Although our analysis was not specifically focused on stromal content or grading of desmoplastic reaction, the strong negative association of DE morphology with survival confirms the biological and clinical relevance of these stromal components. In addition, our findings suggest that PP morphology is associated with improved outcomes, which has not been widely reported in CRC. Given the statistical consistency of this association across both the full and stage I–III cohorts, we believe this signal is unlikely to be spurious. While the biological underpinnings of PP morphology remain underexplored in CRC, it may represent a more differentiated, less invasive phenotype. Further studies are warranted to validate this association and clarify its underlying mechanisms. Lastly, our data support the notion that prognostic associations of morphotypes are not uniform across tumor stages. For example, the protective effect of the CT morphotype was only observed in stage I–III tumors, possibly because in later‐stage disease it co‐occurs with more aggressive components like DE, diluting or masking its individual contribution. This highlights a broader need to move beyond single‐pattern analysis. Given the frequent admixture of morphologies within individual tumors, future work should focus on the combinatorial landscape of morphotypes, which may better reflect tumor biology and its clinical implications. However, such investigations will require larger, well‐annotated cohorts with detailed quantitative morphological data.

In the context of grading, gland formation serves as a central criterion. Consequently, the observation that gland‐rich CT and PP morphotypes are associated with G1 or G2, while TB and, to a lesser extent, MU are associated with G3, is not unexpected. This phenomenon can be interpreted as an internal positive control of the present AI‐morphotype analysis.

Despite being a clonal proliferation of transformed cells, CRC is morphologically strikingly heterogeneous, and the question arises what drives this? Even though we did not perform detailed three‐dimensional mapping of whole tumors in terms of morphotype presence, the visual representation of morphotype distribution in Figure 3E–H is compatible with a dominant clone, corresponding with the dominant morphotype, from which morphologically different subclones arise. This has been suggested before [30]. These subclones would be characterized by additional (epi)genetic events and a specific transcriptome befitting the variant morphology. Additional elements that might contribute to morphological heterogeneity are the tumor microenvironment, which consists of the extracellular matrix, the immune infiltrate, and, more recently, the microbiome. It has been shown, by combining immune gene signatures with microbiome data, that a predictive score can be obtained that performs better than other available predictive biomarkers such as CMS [31]. Evidence in favor of the involvement of the extracellular matrix (ECM) is provided by the three types of DE morphology, with the matrix composition driving cancer cell morphology rather than cancer cells driving matrix composition [32]. CRC tumors with DE morphology with a mature ECM, characterized by fine and well‐aligned fibers stratified in multiple layers, have a better prognosis than tumors with a keloid‐like or myxoid ECM [32]. Therefore, future studies need to also take non‐tumoral elements into account. From this perspective, it would be of great interest to extend the histopathological characterization of intratumoral heterogeneity by inclusion of AI‐based estimates of immune cell distribution within and in close proximity to the tumor area.

It is important to note that our study is not without limitations, and we wish to highlight some intriguing prospective avenues for future study. Firstly, we did not assess the feasibility of utilizing AI‐guided analysis for the characterization of small biopsies. Our current data indicate that this is a feasible approach, as small tumor regions were successfully detected and characterized in our cohort. However, we anticipate an increase in the prevalence of PP and SE morphotypes, as our findings demonstrate a higher frequency of these morphologies in the luminal region, and the biopsies are often superficial. Secondly, we did not analyze the morphological heterogeneity of primary tumor versus matched metastasis. This is a topic of considerable interest that warrants further investigation in future research. Thirdly, we cannot exclude that the total tumor area may affect the degree of heterogeneity, and this point needs to be addressed by performing a per‐area analysis.

In summary, we show that AI‐guided analysis of multiple whole H&E stained sections of a CRC is an appropriate approach to clarify intratumoral heterogeneity, in terms of proportions of morphotypes present and conceivably also in terms of ECM and immune infiltrate. Our data support the notion that spatial quantification of specific morphotypes on multiple slides is likely to be clinically relevant. If the results of this study are validated in additional cohorts, they may have direct implications for the (re)definition of criteria for histological (sub)typing of CRC and the development of evidence‐based protocols for tumor sampling for molecular studies.

## Author contributions statement

EB, VP and MPD conceived and designed the study. RN and MČ performed the sample collection and image data acquisition. RN and FB provided clinical and histopathologic data. MPD, SS and RN performed the histopathological evaluation of the sections. VP performed AI image analysis. EB performed statistical analysis. VP, MPD and EB provided the figures. EB, VP, MPD, DH and FB interpreted the results. MPD and EB drafted the manuscript. MPD, EB, VP and FB performed writing, review and revision of the paper. All authors read, revised and approved the article.

## Supporting information


**Figure S1.** Pairwise analysis of operator agreement by kappa coefficient for individual morphologies
**Figure S2.** Frequency and area of dominant morphotypes across the examined sections and tumors after visual morphotype assessment
**Figure S3.** Frequency of morphotypes in sections with respect to their distribution across the colonic wall
**Table S1.** Additional study population statistics

## Data Availability

The higher resolution H&E images accompanying the examples of the predicted morphotypes (Figure 1B–E) are available from Zenodo: https://doi.org/10.5281/zenodo.14025916. Additional study data can be made available upon reasonable request, subject to data privacy and confidentiality regulations.

## References

[1] Organisation mondiale de la santé, Centre international de recherche sur le cancer . Digestive System Tumours (World Health Organization Classification of Tumours) (5th edn). International Agency for Research on Cancer: Lyon, 2019.

[2] Ono Y , Yilmaz O . Emerging and under‐recognised patterns of colorectal carcinoma morphologies: a comprehensive review. J Clin Pathol 2024; 77: 439–451. 38448211 10.1136/jcp-2023-208816

[3] Dietel M , Jöhrens K , Laffert MV , *et al*. A 2015 update on predictive molecular pathology and its role in targeted cancer therapy: a review focussing on clinical relevance. Cancer Gene Ther 2015; 22: 417–430. 26358176 10.1038/cgt.2015.39

[4] Pang SW , Awi NJ , Armon S , *et al*. Current update of laboratory molecular diagnostics advancement in management of colorectal cancer (CRC). Diagnostics 2019; 10: 9. 31877940 10.3390/diagnostics10010009 PMC7168209

[5] Guinney J , Dienstmann R , Wang X , *et al*. The consensus molecular subtypes of colorectal cancer. Nat Med 2015; 21: 1350–1356. 26457759 10.1038/nm.3967 PMC4636487

[6] Popovici V , Budinská E , Dušek L , *et al*. Image‐based surrogate biomarkers for molecular subtypes of colorectal cancer. Bioinformatics 2017; 33: 2002–2009. 28158480 10.1093/bioinformatics/btx027

[7] Sirinukunwattana K , Domingo E , Richman SD , *et al*. Image‐based consensus molecular subtype (imCMS) classification of colorectal cancer using deep learning. Gut 2021; 70: 544–554. 32690604 10.1136/gutjnl-2019-319866 PMC7873419

[8] Budinská E , Hrivňáková M , Ivkovic TC , *et al*. Molecular portraits of colorectal cancer morphological regions. Elife 2023; 12: RP86655. 37956043 10.7554/eLife.86655 PMC10642970

[9] Budinska E , Popovici V , Tejpar S , *et al*. Gene expression patterns unveil a new level of molecular heterogeneity in colorectal cancer. J Pathol 2013; 231: 63–76. 23836465 10.1002/path.4212 PMC3840702

[10] R Core Team . R: A Language and Environment for Statistical Computing. R Foundation for Statistical Computing: Vienna, 2024.

[11] Whitney‐Miller CL . Impact of subspecialty sign‐out on interobserver variability and accuracy in gastrointestinal pathology. Surg Pathol Clin 2020; 13: 371–376. 32773189 10.1016/j.path.2020.05.001

[12] Shia J , Schultz N , Kuk D , *et al*. Morphological characterization of colorectal cancers in The Cancer Genome Atlas reveals distinct morphology‐molecular associations: clinical and biological implications. Mod Pathol 2017; 30: 599–609. 27982025 10.1038/modpathol.2016.198 PMC5380525

[13] O'Brien MJ , Zhao Q , Yang S . Colorectal serrated pathway cancers and precursors. Histopathology 2015; 66: 49–65. 25263173 10.1111/his.12564

[14] Patankar M , Väyrynen S , Tuomisto A , *et al*. Micropapillary structures in colorectal cancer: an Anoikis‐resistant subpopulation. Anticancer Res 2018; 38: 2915–2921. 29715117 10.21873/anticanres.12539

[15] Kuroda N , Oonishi K , Ohara M , *et al*. Invasive micropapillary carcinoma of the colon: an immunohistochemical study. Med Mol Morphol 2007; 40: 226–230. 18085384 10.1007/s00795-007-0353-z

[16] Remo A , Fassan M , Vanoli A , *et al*. Morphology and molecular features of rare colorectal carcinoma histotypes. Cancer 2019; 11: 1036. 10.3390/cancers11071036 PMC6678907 31340478

[17] Sveen A , Kopetz S , Lothe RA . Biomarker‐guided therapy for colorectal cancer: strength in complexity. Nat Rev Clin Oncol 2020; 17: 11–32. 31289352 10.1038/s41571-019-0241-1 PMC7577509

[18] Büttner J , Jöhrens K , Klauschen F , *et al*. Intratumoral morphological heterogeneity can be an indicator of genetic heterogeneity in colorectal cancer. Exp Mol Pathol 2018; 104: 76–81. 29337243 10.1016/j.yexmp.2018.01.007

[19] Roepman P , Schlicker A , Tabernero J , *et al*. Colorectal cancer intrinsic subtypes predict chemotherapy benefit, deficient mismatch repair and epithelial‐to‐mesenchymal transition. Int J Cancer 2014; 134: 552–562. 23852808 10.1002/ijc.28387 PMC4234005

[20] Trinh A , Trumpi K , De Sousa E Melo F , *et al*. Practical and robust identification of molecular subtypes in colorectal cancer by immunohistochemistry. Clin Cancer Res 2017; 23: 387–398. 27459899 10.1158/1078-0432.CCR-16-0680

[21] de Sousa E Melo F , Wang X , Jansen M , *et al*. Poor‐prognosis colon cancer is defined by a molecularly distinct subtype and develops from serrated precursor lesions. Nat Med 2013; 19: 614–618. 23584090 10.1038/nm.3174

[22] Song N , Pogue‐Geile KL , Gavin PG , *et al*. Clinical outcome from oxaliplatin treatment in stage II/III colon cancer according to intrinsic subtypes: secondary analysis of NSABP C‐07/NRG oncology randomized clinical trial. JAMA Oncol 2016; 2: 1162–1169. 27270348 10.1001/jamaoncol.2016.2314 PMC5065181

[23] Minciuna CE , Tanase M , Manuc TE , *et al*. The seen and the unseen: molecular classification and image based‐analysis of gastrointestinal cancers. Comput Struct Biotechnol J 2022; 20: 5065–5075. 36187924 10.1016/j.csbj.2022.09.010 PMC9489806

[24] Wood CS , Pennel KAF , Leslie H , *et al*. Spatially resolved transcriptomics deconvolutes prognostic histological subgroups in patients with colorectal cancer and synchronous liver metastases. Cancer Res 2023; 83: 1329–1344. 37057593 10.1158/0008-5472.CAN-22-2794 PMC10102851

[25] Wang F , Long J , Li L , *et al*. Single‐cell and spatial transcriptome analysis reveals the cellular heterogeneity of liver metastatic colorectal cancer. Sci Adv 2023; 9: eadf5464. 37327339 10.1126/sciadv.adf5464 PMC10275599

[26] Qi J , Sun H , Zhang Y , *et al*. Single‐cell and spatial analysis reveal interaction of FAP+ fibroblasts and SPP1+ macrophages in colorectal cancer. Nat Commun 2022; 13: 1742. 35365629 10.1038/s41467-022-29366-6 PMC8976074

[27] Green JB , Timmcke AE , Mitchell WT , *et al*. Mucinous carcinoma – just another colon cancer? Dis Colon Rectum 1993; 36: 49–54. 8380140 10.1007/BF02050301

[28] Hu Q , Wang Y , Yao S , *et al*. Desmoplastic reaction associates with prognosis and adjuvant chemotherapy response in colorectal cancer: a multicenter retrospective study. Cancer Res Commun 2023; 3: 1057–1066. 37377615 10.1158/2767-9764.CRC-23-0073 PMC10269709

[29] Ueno H , Ishiguro M , Nakatani E , *et al*. Prognostic value of desmoplastic reaction characterisation in stage II colon cancer: prospective validation in a phase 3 study (SACURA trial). Br J Cancer 2021; 124: 1088–1097. 33414540 10.1038/s41416-020-01222-8 PMC7960987

[30] Baisse B , Bouzourene H , Saraga EP , *et al*. Intratumor genetic heterogeneity in advanced human colorectal adenocarcinoma. Int J Cancer 2001; 93: 346–352. 11433398 10.1002/ijc.1343

[31] Roelands J , Kuppen PJK , Ahmed EI , *et al*. An integrated tumor, immune and microbiome atlas of colon cancer. Nat Med 2023; 29: 1273–1286. 37202560 10.1038/s41591-023-02324-5 PMC10202816

[32] Ueno H , Jones A , Jass JR , *et al*. Clinicopathological significance of the ‘keloid‐like’ collagen and myxoid stroma in advanced rectal cancer. Histopathology 2002; 40: 327–334. 11943016 10.1046/j.1365-2559.2002.01376.x

