# Supplementary material for: A quantitative tumor‐wide analysis of morphological heterogeneity of colorectal adenocarcinoma
Source: J Pathol Clin Res. 2025 Jun 13;11(4):e70034. doi: 10.1002/2056-4538.70034 (PMC12163513; doi:10.1002/2056-4538.70034)
Supplement: Supplementary file 1 — Figure S1. Pairwise analysis of operator agreement by kappa coefficient for individual morphologies Figure S2. Frequency and area of dominant morphotypes across the examined sections and tumors after visual morphotype assessment Figure S3. Frequency of morphotypes in sections with respect to their distribution across the colonic wall Table S1. Additional study population statistics [file CJP2-11-e70034-s001.pdf]

A quantitative tumor-wide analysis of morphological heterogeneity of colorectal adenocarcinoma

MP Dragomir *et al.*, *J Pathol Clin Res*, https://doi.org/10.1002/2056-4538.70034

Supplementary Figures S1–S3
Supplementary Table S1

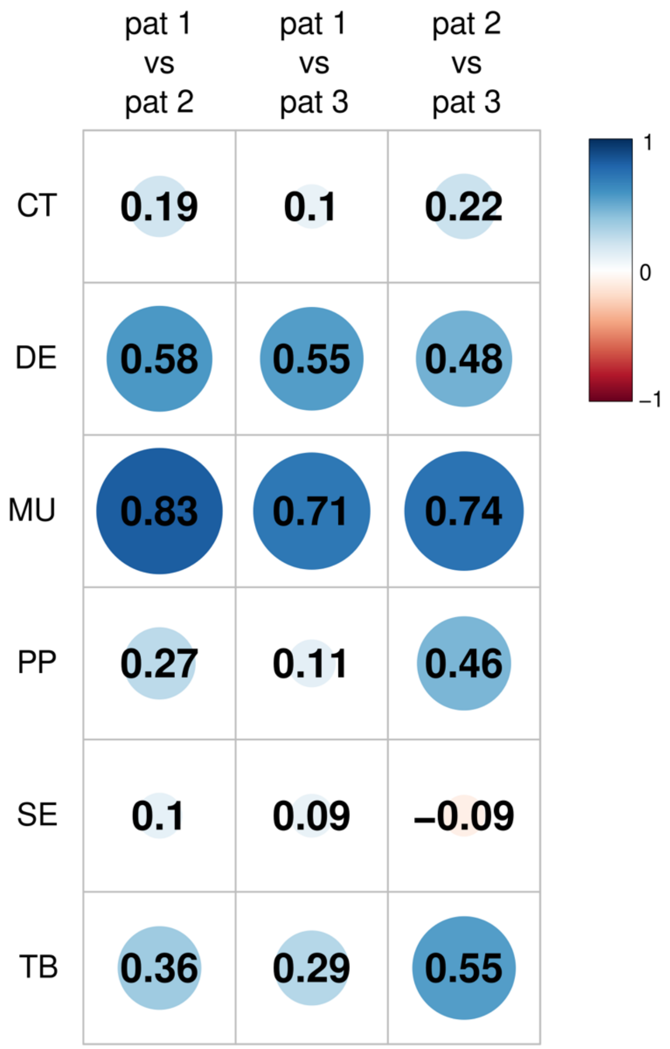

Figure S1. Pairwise analysis of operator agreement by kappa coefficient for individual morphologies, where values represent weak (0.4–0.59), moderate (0.6–0.79), strong (0.8–0.9), and almost perfect (>0.9) agreement.

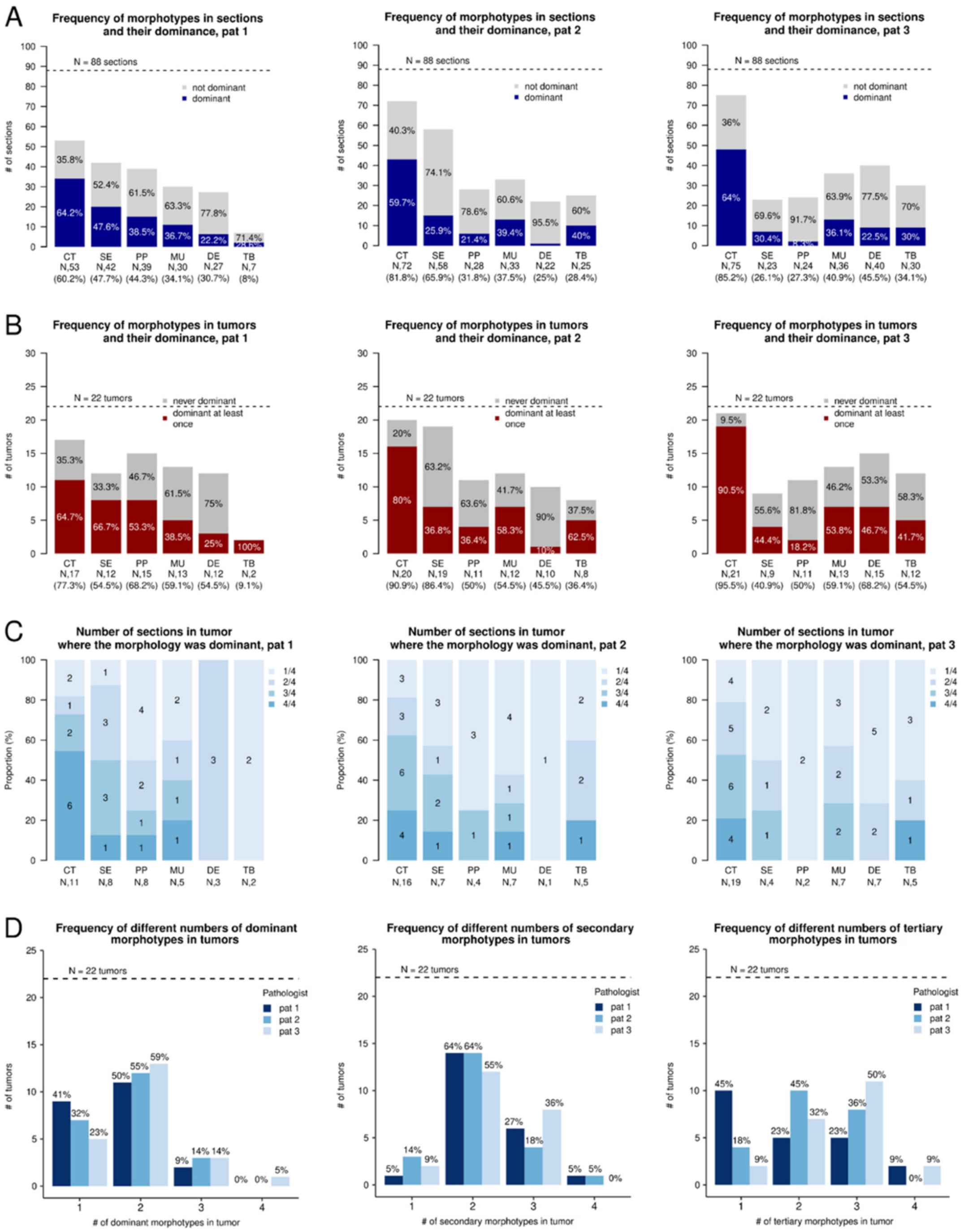

Figure S2. Frequency and area of dominant morphotypes across the examined sections and tumors after visual morphotype assessment. (**A**) Frequency of morphotypes in sections with respect to their dominance. (**B**) Frequency of morphotypes in tumors with respect to their dominance. (**C**) Frequency of sections in tumors where the morphology was dominant. 1/4 means the morphotype was dominant in one of the four examined slides of the tumor, etc. (**D**) Frequency of different numbers of dominant (left), secondary (middle), and tertiary (right) morphotypes across the four examined sections, as evaluated by three expert pathologists.

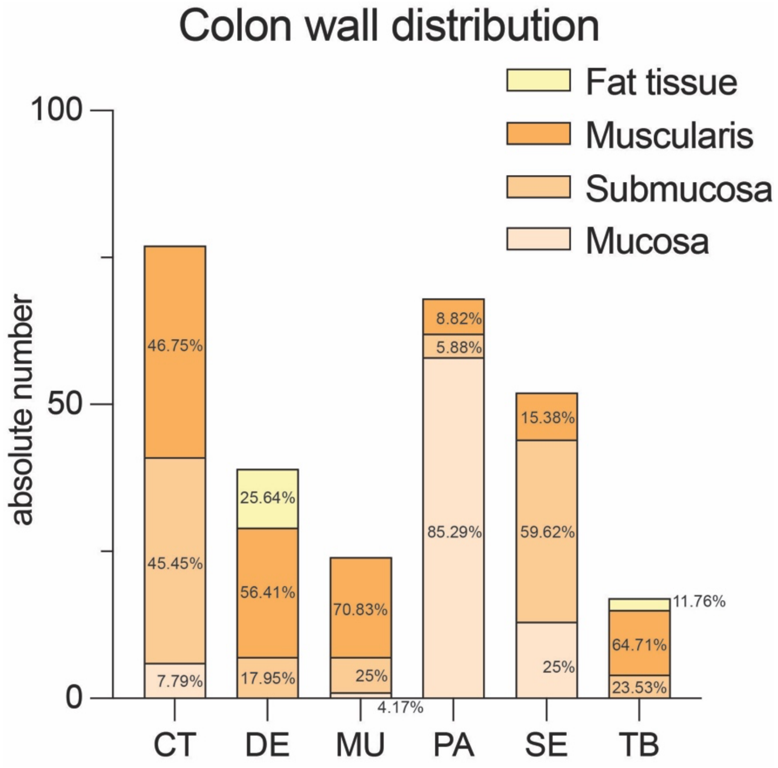

Figure S3. Frequency of morphotypes in sections with respect to their distribution across the colonic wall (mucosa, submucosa, muscularis propria, and fat tissue). For statistical analysis we compared the frequency of morphotypes in sections with respect to their distribution across the colonic wall in a binary analysis of superficial (mucosa and submucosa) versus deep (muscularis propria and fat tissue).

Table S1 Additional study population statistics.

**Stratified by gender**				
	**F**	**M**	***p***	***p*-adj**
n	**72**	**89**		
CT (median [IQR])	44.79 [18.03, 62.44]	46.79 [26.75, 60.70]	0.484	0.692
DE (median [IQR])	5.53 [0.00, 12.69]	2.60 [0.00, 9.30]	0.069	0.144
MU (median [IQR])	0.00 [0.00, 7.24]	1.32 [0.00, 7.33]	0.668	0.828
PP (median [IQR])	12.63 [5.63, 26.66]	16.87 [9.38, 32.85]	0.075	0.145
SE (median [IQR])	4.94 [1.80, 13.82]	5.50 [1.75, 10.63]	0.799	0.918
**TB (median [IQR])**	**2.90 [0.00, 10.88]**	**0.00 [0.00, 2.74]**	**0.003**	**0.009**

**Stratified by AJCC stage**						
	**I**	**II**	**III**	**IV**	***p***	***p*-adj**
n	**24**	**67**	**43**	**27**		
CT (median [IQR])	46.90 [35.32, 65.42]	44.84 [20.63, 61.90]	50.68 [13.69, 59.29]	43.00 [30.36, 60.02]	0.929	0.949
**DE (median [IQR])**	**0.00 [0.00, 3.09]**	**3.53 [0.00, 8.66]**	**5.10 [0.00, 14.63]**	**10.28 [4.08, 24.30]**	**<0.001**	**<0.001**
MU (median [IQR])	0.00 [0.00, 3.73]	1.33 [0.00, 12.63]	1.57 [0.00, 10.46]	0.00 [0.00, 4.65]	0.466	0.692
PP (median [IQR])	20.30 [10.06, 42.55]	17.19 [9.22, 29.87]	13.10 [6.60, 26.21]	8.87 [5.14, 21.14]	0.046	0.110
SE (median [IQR])	5.98 [1.63, 16.95]	5.55 [2.31, 12.71]	5.06 [1.85, 9.46]	4.19 [0.00, 12.32]	0.838	0.924
TB (median [IQR])	0.65 [0.00, 5.04]	0.00 [0.00, 6.62]	0.00 [0.00, 7.19]	1.29 [0.00, 5.33]	0.996	0.996

**Stratified by grade**					
	**1**	**2**	**3**	***p***	***p*-adj**
n	**19**	**95**	**47**		
**CT (median [IQR])**	**56.93 [19.71, 61.70]**	**52.01 [38.28, 66.80]**	**23.33 [8.82, 43.43]**	**<0.001**	**<0.001**
DE (median [IQR])	3.64 [0.00, 9.80]	3.66 [0.00, 10.60]	4.30 [0.00, 12.83]	0.519	0.692
**MU (median [IQR])**	**0.00 [0.00, 6.73]**	**0.00 [0.00, 4.42]**	**3.81 [0.00, 38.09]**	**0.026**	**0.073**
**PP (median [IQR])**	**17.66 [11.10, 41.91]**	**16.13 [10.10, 32.60]**	**6.14 [1.56, 22.13]**	**<0.001**	**0.002**
SE (median [IQR])	8.40 [4.95, 13.34]	5.28 [1.80, 11.67]	3.91 [0.00, 12.25]	0.060	0.132
**TB (median [IQR])**	**0.00 [0.00, 0.00]**	**0.00 [0.00, 4.49]**	**3.20 [0.00, 27.86]**	**0.001**	**0.006**

Stratified by site

	Right	Transverse	Left	Rectosigmoid	Rectum	*p*	*p*-adj
n	**55**	**18**	**44**	**27**	**17**		
**CT (median [IQR])**	**24.62 [12.69, 42.92]**	**48.56 [26.82, 65.46]**	**49.16 [39.79, 62.50]**	**59.51 [44.31, 73.78]**	**59.32 [47.16, 64.91]**	**<0.001**	**<0.001**
DE (median [IQR])	4.73 [1.43, 14.31]	6.69 [0.53, 9.11]	1.49 [0.00, 8.26]	1.69 [0.00, 8.54]	3.66 [0.00, 14.01]	0.178	0.329
**MU (median [IQR])**	**4.94 [1.69, 20.55]**	**0.00 [0.00, 1.79]**	**0.00 [0.00, 2.43]**	**0.00 [0.00, 2.11]**	**0.00 [0.00, 1.32]**	**<0.001**	**<0.001**
PP (median [IQR])	12.51 [4.80, 32.88]	12.58 [4.07, 30.99]	19.74 [9.93, 34.19]	12.39 [7.86, 19.52]	15.17 [13.10, 23.44]	0.379	0.674
SE (median [IQR])	5.74 [1.65, 13.97]	4.16 [1.39, 11.80]	6.02 [1.98, 13.02]	5.28 [2.84, 12.00]	2.72 [0.00, 10.63]	0.858	0.924
**TB (median [IQR])**	**1.30 [0.00, 13.59]**	**4.22 [0.00, 9.95]**	**0.00 [0.00, 4.49]**	**0.00 [0.00, 3.20]**	**1.29 [0.00, 3.51]**	**0.038**	**0.096**

Stratified by pT

	T1	T2	T3	T4	*p*	*p*-adj
n	**8**	**25**	**115**	**13**		
CT (median [IQR])	47.81 [45.98, 53.60]	47.36 [32.29, 66.98]	44.75 [18.84, 60.67]	52.01 [29.38, 66.62]	0.578	0.750
**DE (median [IQR])**	**0.00 [0.00, 0.00]**	**1.39 [0.00, 7.01]**	**4.11 [0.00, 11.18]**	**12.67 [4.46, 25.44]**	**0.001**	**0.005**
MU (median [IQR])	0.00 [0.00, 0.00]	1.57 [0.00, 4.83]	1.49 [0.00, 9.67]	0.00 [0.00, 10.13]	0.059	0.132
**PP (median [IQR])**	**28.81 [21.54, 42.55]**	**15.17 [9.53, 40.71]**	**14.47 [7.90, 28.55]**	**3.48 [1.66, 5.00]**	**<0.001**	**<0.001**
SE (median [IQR])	2.41 [1.21, 15.97]	8.13 [2.03, 15.23]	5.55 [1.87, 12.71]	1.90 [0.00, 3.65]	0.073	0.145
TB (median [IQR])	0.00 [0.00, 4.62]	0.00 [0.00, 4.91]	0.00 [0.00, 6.54]	3.12 [0.00, 6.69]	0.803	0.918

Stratified by pN

	N0	N1	N2	*p*	*p*-adj
n	**94**	**41**	**26**		
CT (median [IQR])	45.80 [21.50, 63.39]	47.38 [27.79, 59.26]	40.31 [10.68, 59.46]	0.474	0.692
**DE (median [IQR])**	**2.02 [0.00, 7.63]**	**5.10 [0.00, 19.09]**	**9.90 [5.15, 19.99]**	**<0.001**	**<0.001**
MU (median [IQR])	0.00 [0.00, 5.88]	1.30 [0.00, 10.29]	3.10 [0.00, 6.58]	0.673	0.828
**PP (median [IQR])**	**17.73 [10.06, 34.11]**	**12.67 [6.88, 24.62]**	**8.49 [3.86, 14.04]**	**0.012**	**0.036**
SE (median [IQR])	5.85 [1.98, 13.01]	5.59 [1.79, 10.31]	3.16 [0.00, 13.16]	0.458	0.692
TB (median [IQR])	0.00 [0.00, 5.97]	0.00 [0.00, 4.31]	0.64 [0.00, 10.28]	0.782	0.918

**Stratified by pM**				
	**M0**	**M1**	***p***	***p*-adj**
n	**134**	**27**		
CT (median [IQR])	46.71 [20.82, 61.93]	43.00 [30.36, 60.02]	0.885	0.923
**DE (median [IQR])**	**2.76 [0.00, 9.28]**	**10.28 [4.08, 24.30]**	**<0.001**	**0.002**
MU (median [IQR])	1.32 [0.00, 9.86]	0.00 [0.00, 4.65]	0.482	0.692
**PP (median [IQR])**	**15.45 [8.43, 31.86]**	**8.87 [5.14, 21.14]**	**0.032**	**0.086**
SE (median [IQR])	5.47 [1.90, 12.77]	4.19 [0.00, 12.32]	0.490	0.692
TB (median [IQR])	0.00 [0.00, 6.43]	1.29 [0.00, 5.33]	0.884	0.923

**Stratified by MSI**				
	**MSI**	**MSS**	***p***	***p*-adj**
n	**104**	**27**		
**CT (median [IQR])**	**17.92 [8.82, 31.74]**	**53.35 [35.76, 65.77]**	**<0.001**	**<0.001**
DE (median [IQR])	4.30 [0.00, 12.06]	2.61 [0.00, 10.12]	0.510	0.692
**MU (median [IQR])**	**12.95 [0.00, 42.30]**	**0.00 [0.00, 3.30]**	**<0.001**	**0.0015**
**PP (median [IQR])**	**8.49 [1.43, 14.44]**	**15.17 [9.14, 28.32]**	**0.002**	**0.007**
SE (median [IQR])	4.68 [0.00, 12.38]	5.04 [1.79, 12.65]	0.498	0.692
**TB (median [IQR])**	**12.21 [0.00, 41.67]**	**0.00 [0.00, 3.22]**	**<0.001**	**<0.001**
